# Supplementary material for: Occurrence and diversity of Campylobacter species in diarrheic children and their exposure environments in Ethiopia
Source: PLOS Glob Public Health. 2024 Oct 29;4(10):e0003885. doi: 10.1371/journal.pgph.0003885 (PMC11521251; doi:10.1371/journal.pgph.0003885)
Supplement: Attachment — Submitted filename: PGPH-D-24-01065 Detection of Campy in the Envt_AAM.docx [file pgph.0003885.s001.docx]

**Detection and Occurrence Patterns of *Campylobacter* species in the Exposure Environments of Diarrheic Children in Ethiopia**

*Dinaol Belina^1,3^, Tesfaye Gobena^2^, Ameha Kebede^3^, Meseret Chimdessa^3^, Tine Hald^4^

^1^College of Veterinary Medicine, Haramaya University, P.O. Box 138, Dire Dawa, Ethiopia

^2^College of Health and Medical Sciences, Haramaya University, Harar, Ethiopia

^3^School of Biological Sciences and Biotechnology, Haramaya University, Dire Dawa, Ethiopia

**^4^**National Food Institute, Technical University of Denmark, Lyngby, Denmark

*Corresponding author: [belina.dinaol@gmail.com](mailto:belina.dinaol@gmail.com)

**Abstract**

*Campylobacter* is an important public health concern pathogen, particularly in children and immune compromised individuals. Infected as well as apparently healthy humans and animals, contaminated food, and the environment, can all act as sources of this pathogen. This study was aimed at estimating the prevalence and monthly occurrence patterns, and identifying the specific *Campylobacter* spp. circulating in the physical exposures of diarrheic children in urban and rural settings in Ethiopia. **Materials and Methods:** Through case-based tracking, a total of 438 exposure sources, including animal, food, and environmental samples, were collected from Harar town and Kersa district between November 2021 and January 2023. A standard laboratory technique was used to identify Campylobacter using selective media. The identified isolates were confirmed with qPCR, followed by sequencing, and bioinformatic tools were employed for species identification. **Results:** The overall prevalence of *Campylobacter* in the exposure sources was found to be 5.5%, with 6% and 5% in urban and rural settings, respectively. The study identified only *C. jejuni* and *C. coli,* with *C. jejuni* generally being the more frequently identified *Campylobacter* spp. The study also found fluctuations in the monthly occurrence patterns of *Campylobacter,* with the highest monthly occurrence of 20.7% observed in February 2022. *Campylobacter* detection was 1.8 times more likely in household samples (8.7%; OR = 1.8; 95% CI: 0.68–4.52) than in samples collected from marketplaces linked to diarrheic cases. The occurrence of *Campylobacter* in food was 4.2%, with no significant differences observed among meat, milk, and other food categories. On the other hand, logistic regression analysis revealed that the likelihood of *Campylobacter* spp. contamination in the environment was 5.8 times higher in the presence of poultry feces (17.7%; OR = 5.8; CI: 1.1–30.6) compared to shoat feces. Interestingly, *Campylobacter* was not detected in water sources. **Conclusion**: In conclusion, *Campylobacter* was detected in various samples in the exposure environments of diarrheic cases, with no significant differences in its occurrence between Kersa and Harar town, as well as among meat, milk, and other food categories, demonstrating it is equally important in all food types in both rural and urban settings. A genome-based integrated study at the human-animal-environment interface is recommended to determine the relative contribution of each source.

***Keywords***: Diarrheic children, Exposures sources, Foodborne pathogen, One health, Ethiopia

**Introduction**

*Campylobacter* is a bacterium that commonly inhabits the gastrointestinal tract of most warm-blooded animals. It is one of the most common causes of bacterial gastroenteritis and foodborne diseases (FBD) globally. Campylobacteriosis, a disease caused by *Campylobacter* spp., is a zoonotic disease and has been documented as causing diarrhea in both human and animal populations across the globe. Recently, *Campylobacter* has been recognized as one of the four key global causes of diarrheal diseases that greatly contributes to the burden of FBD, particularly in developing countries (1–3).

*Campylobacter* spp., like other enteric pathogens, is primarily transmitted through the fecal-oral route, which occurs when individuals ingest contaminated food or water. Infected humans, animals, and their environment can all act as sources of this pathogen. Apparently healthy humans and animals, especially chickens*,* can also be sources of *Campylobacter* as they can carry the pathogen without any noticeable symptoms. However, the occurrence and transmission of *Campylobacter*-associated diarrheal disease are influenced by various risk factors. These include poor sanitation, inadequate access to safe drinking water, socioeconomic status, behavioral aspects, etc. Specifically, the consumption of raw or undercooked poultry, meat, or unpasteurized dairy products increases the risk of *Campylobacter* infection (4–6). Another risk factor associated with enteric pathogen infection is the season of the year. Campylobacter infection occurs year-round in low- and middle-income countries and exhibits late summer and fall seasonality in developed countries (2,7).

*Campylobacter* induced diarrheal infection is a major public health concern in low-income countries, particularly in children under the age of five (UFC) and elderly individuals (1). In Ethiopia, *Campylobacter* spp. have been documented in diarrheic UFC cases at varying levels of occurrence, ranging from 6.8% (3) to 15.2% (8). Being in close proximity to carrier animals can increase the chances of contracting the disease and its prevalence. For instance, a study carried out in Ethiopia has identified that coming into contact with animals, particularly chickens, as a major risk factor for *Campylobacter*-associated diarrhea in children (9). There have been multiple research studies highlighting the importance of *Campylobacter* in food (7,10,11) in Ethiopia, with prevalence rates reaching up to 56.5% in animals(12)13% in poultry, and 10.5% in water (13). Like other foodborne pathogens, the health impacts of *Campylobacter* is further aggravated in rural areas (14).

The residents in different villages in eastern Ethiopia have limited awareness about zoonotic diseases and do not practice proper hand hygiene (15,16). Moreover, open defecation is common in these areas (17). Consequently, *Campylobacter* and other diarrheagenic pathogens may be more prevalent in the environment in areas such as Harar and Kersa district. Despite the high risk of contracting and transmitting zoonotic diseases, a compressive study on the occurrence of *Campylobacter* in asymptomatic humans, animals, and the environment in the exposure environment of diarrheic cases is generally missing in Ethiopia. Previous studies conducted on *Campylobacter* or other diarrheagenic FBPs have focused on their prevalence in diarrheic human cases, asymptomatic food handlers, animals**, or** food and environmental samples separately.

Furthermore, there is a scarcity of data on the monthly patterns of *Campylobacter* occurrence and the differences between urban and rural settings in Ethiopia. As a result, it is crucial to explore the occurrence of *Campylobacter* at the human-animal-environment interface to develop effective prevention measures. Therefore, the aim of this study was to identify the most common *Campylobacter* species, and estimate the prevalence and monthly occurrence patterns of Campylobacter in general in animal, food, and environmental samples in the immediate environments of diarrheic children in both urban and rural settings in Ethiopia.

**Material and Methods**

**Study area and Sample types**

A case-based contact tracing was undertaken in Harar town and Kersa district, eastern Ethiopia. (18). Only samples from children whose families reported direct or indirect contact with the sample(s) shortly before the onset of diarrheal symptoms and signs were included in the study. Specifically, through case-based tracking, various environmental samples including animal feces, different food items, water, and wastewater samples were collected from the immediate physical environments of the known diarrheic UFC.

**Sampling Techniques and Procedures**

Before tracking the environmental samples, the diarrheic UFC cases living in Harar town and Kersa district, Ethiopia, were screened and identified as outlined in our published article (18). Following the case identification, a face-to-face interview was conducted with caretakers to obtain relevant background and the case’s recent exposure details. These include a history of what the child has ingested, the origin of the consumed food (self-produced, purchased from market, etc.), information about animal contact, specific household addresses, etc. The interview questionnaire was also used to identify the source of drinking water, irrigation water, or recreational water used prior to the onset of diarrheal symptoms. Again, the possible exposure to wastewater linked to households or any sites was also assessed through interviews with caretakers (**Figure 1**).

**Collection of exposure samples from the households,** **food production sites and marketplaces**: Following the interview, the caretakers were requested for their agreement to visit households, and other identified contact sites for sample collections. Those visits were made within 1 to 2 days after the collection of diarrheic stool samples. Guided by the caretakers’ responses during the interviews, animal, food, water source and wastewater samples were collected from sites where the case had recent physical exposure and indirect contact. The questionnaire responses used as guidance for the sample collection process were initially filled on a tablet using Epi Info 7 software at the health facilities, and allowed us to identify specific locations and types of samples to be collected before going for visits. The identified physical exposure environments considered in this study were households, nearby farms, lakes, local marketplaces, hotels or restaurants, butcher shops, and slaughterhouse facilities where a case or a case's family member(s) had direct or indirect contact.

The collected food samples were animal-sourced foods–such as raw bovine, camel, sheep, and goat meats, chicken, raw milk, and eggs; non-animal-origin foods such as vegetables, fruit, and imported as well as locally prepared juices, and, other ready-to-eat (RTE) foods, including mixed food items like "injera with wot". Drinking water, recreational water, and irrigation water samples were also collected from various sources. Moreover, feces were collected aseptically from any animal species in the households, such as cattle, camels, sheep, goats, and poultry. The fecal samples were collected either directly from the rectum or from fresh droppings. In cases where multiple animals of the same species were present in households, only pooled fecal samples were collected from them. Feces from live or newly slaughtered animals at abattoir and marketplaces were also tracked.

We also collected wastewater runoff from slaughterhouses (animal wastewater) or food preparation sites (mixed human and animal wastewater) and wastewater from humans only (e.g., from utensils and handwashing at hotels or restaurants linked to the case or wastewater discharged from households.

Tracking and collection of various environmental samples, including food, animal, wastewater and water samples

UFC with diarrhea

Residents of Harar town or Kersa district

Informed assents to sample of caregiver

No Household visits

Informed consents, to sample of UFC and face to face interview

Collection of UFC stool and Interview with Caregiver

Appointment or arranging date/time for household visit, hands over leftover food and feces collection kit to caregiver with verbal and written instruction

Marketplace visits

Yes

No

Exclude

No

Exclude

No

Exclude

No

Yes

Informed consents/assents to sample of caregiver, possible sibling and household visit

Yes

Yes

No

Yes

Collection of caregiver feces

Household visits

Screening was made against Inclusion and Exclusion Criteria

Food production sites, etc. visits

**Figure 1** Procedures used for case identification and collection of environmental samples

**Sample Transportation, preparation, and Isolation and identification of *Campylobacter***

All samples were aseptically collected into sterile stomacher bags (Fisher Scientific Biotech line, Cat. 10576064) from the identified sites, labeled and promptly placed in a cold box with ice packs for transportation on the same day to Haramaya University, the Microbiology Laboratory, Harar. The sample preparation adhered to a specific Standard Operating Procedure (SOP) developed for the FOCAL project, with minor modifications to the previously described ISO 10272-1 protocol (19,20).

The samples underwent gentle mixing before suspending one gram of animal feces and either 10 grams of food or 10 mL of liquid samples in 9 mL and 90 mL of bolton broth, respectively, using a 1:9 (v/v) sample to bolton broth ratio. On the other hand, non-turbid water, like drinking water, was prepared in a 1:1 (v/v) sample to broth ratio. The samples were then microaerophilically incubated at 37°C for 4 hours, followed by incubation at 42°C for 44±4 hours. After a 48-hour incubation period, a loop full of suspension from the *Campylobacter* selective enrichment, bolton broth, was plated on blood-free *Campylobacter* selective agar (mCCDA-modified charcoal cefoperazone deoxycholate agar), and the isolation and identification of *Campylobacter* spp. were conducted as previously described (18,21).

***Campylobacter* Confirmation and Species identification**

Genomic DNA was extracted from pure bacterial cultures (Figure 3A) through thermal lysis, as previously outlined (22). The concentration and purity of the DNA were assessed spectrophotometrically using a NanoPhotometer (Mettler-Toledo (S) Pte Ltd., Singapore). All samples with an A_260_/A_280_ ratio of 1.7 or above were considered for further analysis (Figure 3B), while those below 1.7 were re-cultured to improve the DNA purity. The extracted DNAs were used for qPCR genus-level *Campylobacter* confirmation as previously described (23) with GATGACACTTTTCGGAGCGTAA and GCTTGCACCCTCCGTATTA as the forward and reverse primers, respectively, in accordance with the ZymoScript™ One-Step RT-qPCR Kit protocol (24). The qPCR-confirmed *Campylobacter* isolates were then subcultured on Brain Heart Infusion (BHI) Agar and underwent DNA extraction using the Quick-DNA™ Fungal/Bacterial Miniprep Kit (25) for further analysis. Subsequently, 73% (37 out of 51) of the DNA samples were subjected to species identification through whole genome sequencing at the Genohub Project, Admera Health, LLC, USA.

Data quality Control

Throughout the study, data quality was maintained by adhering to a SOP specifically developed for the FOCAL project. Quality controls for the bacteriology laboratory activities were ensured as described before (18). The Whole-Genome Sequencing (WGS) reads from each sample were trimmed and quality assessed using the bioinformatic tools FASTQC and bbduk2 for the species identification.

Data management and Analysis

The laboratory data was entered into Microsoft Excel, cross checked, and exported into Statistical Package for the Social Sciences (SPSS) version 22 for analysis. Descriptive statistics were used to calculate and interpret the study variables. To determine the prevalence of *Campylobacter*, the total number of positive samples was divided by the total number of samples processed in the laboratory, multiplied by 100. The Pearson chi-square (X^2^) test was used to assess the association between explanatory variables, and observed differences were considered statistically significant when 𝑃 value ≤ 0.05. The monthly *Campylobacter* occurrence rate was calculated by considering the number of *Campylobacter* isolates obtained in a given month and the total number of samples analysed in the laboratory in that particular month. Moreover, binary logistic regression model was executed and the odds ratio was employed to measure the association between independent and dependent variables. The *Speciesfider* and KMA (*k*mer alignment) tools, accessed from the Center for Genomic Epidemiology (CGE) page (<https://www.genomicepidemiology.org/services/>), were used to determine the species of the *Campylobacter* isolates.

**Ethical considerations and participants’ consents**

The study received ethical approval from the Institutional Health Research Ethics Review Committee (IHRERC) and the National Research Ethics Committee, Ethiopia, through a letter dated February 11, 2020 (Ref Number: MoSHE/RD/14.1/9849/20). The caregivers were fully informed about the study and were requested to provide their informed consent in order to take part. Stool samples were collected from diarrheic UFC at healthcare facilities upon a routine stool test request for treatment, as well as from their caretaker and youngest sibling, with verbal consent from caretakers. Caregivers also agreed to the collection of possible contact samples from the immediate exposure environments of the diarrheic child. Throughout the study, the confidentiality of the participants' information, as well as that of their parents or caregivers, was strictly maintained.

**Results**

In this study, the occurrence of *Campylobacter* spp. in the exposure environment of diarrheic children was investigated by analysing the animal, food, and environmental samples in the vicinity. The overall prevalence of *Campylobacter* was found to be 6.5% (51/783), with 5.5% in the exposure environments of the cases. However, the occurrence of *Campylobacter* had no significant difference among cases, caretakers, and environmental samples (**Table 1**). Out of the 37 samples analysed for species identification, it was found that *C. jejuni* and *C. coli* were the only species detected, with a frequency rate of 62.16% and 37.84%, respectively. Among the 20 sequenced environmental isolates, half of them (50%) were identified as *C. jejuni*. The distribution of *Campylobacter* spp. across sample types in eastern Ethiopia is presented in **Table 2**.

**Table 1** Overall prevalence of *Campylobacter* spp. in the exposure environments of diarrheic children in eastern Ethiopia**.**

| Sample Type | Total sample | Positive (%) | X^2^ (P value) |
| --- | --- | --- | --- |
| Environmental | 438 | 24(5.48) | 2.4(0.31) |
| Caretaker | 83 | 5(6.02)** |  |
| UFC | 262 | 22(8.4)** |  |
| Total | **783** | **51(6.51)** |  |

** Full data accessed in: Belina *et al.,* 2023; <https://pubmed.ncbi.nlm.nih.gov/37736061/>

**Table 2** Frequency of distribution of *Campylobacter* spp. detected in diarrheic UFC and their exposure environments in eastern Ethiopia**.**

| Sample type | Frequency (%) |  | Total |
| --- | --- | --- | --- |
|  | ***C. jejuni*** | ***C. coli*** |  |
| Environmental | 10(50) | 10(50) | 20(54.05) |
| Caretaker ** | 3(75) | 1(25) | 4(10.81) |
| Diarrheic UFC ** | 10(76.92) | 3(23.08) | 13(35.14) |
| Total | **23(62.16)** | **14(37.84)** | **37(100)** |

The prevalence of *Campylobacter* spp. in samples collected from abattoirs, households and marketplaces was 3.8%, 8.7%, and 5.1%, respectively. Furthermore, our study showed that *Campylobacter* spp. was detected in 6.7% of animal faces (6.7%, OR = 0.72; 95% CI: 0.25–2.03) and 4.2% of food samples (4.2%, OR = 0.44; 95% CI: 0.14–1.35). Nevertheless, *Campylobacter* was not detected in water samples collected from various sources. Interestingly, the occurrence of *Campylobacter* did not show statistical differences across sample types and sources, as well as between the rural and urban settings (OR=1.3; 95% CI: 0.53–2.78) of the study (**Table 3).**

**Table 3** Occurrence of *Campylobacter* spp. in environmental samples collected from different sources in Kersa and Harar, Ethiopia (N=438)

| Samples Source and Type | | Negative | Positive | OR | 95% CI | P value |
| --- | --- | --- | --- | --- | --- | --- |
| Source | Abattoir | 127(96.21) | 5(3.79) | 0.73 | 0.25–2.14 | 0.56 |
|  | Household | 84(91.3) | 8(8.7) | 1.8 | 0.68–4.52 | 0.24 |
|  | Market | 203(94.86) | 11(5.14) | Ref | Ref |  |
| Type | Animal faces | 153(93.29) | 11(6.71) | 0.72 | 0.25–2.03 | 0.53 |
|  | Food | 161(95.83) | 7(4.17) | 0.44 | 0.14–1.35 | 0.15 |
|  | Wastewater | 60(90.91) | 6(9.09) | Ref | Ref |  |
|  | Water sources | 40(100) | 0(0) | – | – |  |
| Study area | Harar town | 204(94.01) | 13(5.99) | 1.3 | 0.53–2.78 | 0.64 |
|  | Kersa district | 210(95.02) | 11(4.98) | Ref | Ref |  |
|  | **Total** | **414(94.52)** | **24(5.48)** | – | – |  |

Water sources–Drinking, Recreational and Irrigation water

The study revealed that there were fluctuations in the monthly occurrence patterns of *Campylobacter* spp. throughout the study period, spanning from November 2021 to January 2023, reaching a peak in February 2022. Generally, the occurrence rate was notably higher during the winter months, with a distinct linearly increasing pattern observed from November 2021 to February 2022. The second highest monthly occurrence was observed during the rainy season, with a peak of 6.7% in July 2022 (**Figure 2**).

|  |  |
| --- | --- |
| 2021-11-01 | 9.52381 |
| 2021-12-01 | 10.4651 |
| 2022-01-01 | 15.3846 |
| 2022-02-01 | 20.6897 |
| 2022-03-01 | 5.81395 |
| 2022-04-01 | 4.25532 |
| 2022-05-01 | 3.27869 |
| 2022-06-01 | 2.5 |
| 2022-07-01 | 6.66667 |
| 2022-08-01 | 2.27273 |
| 2022-09-01 | 3.57143 |
| 2022-10-01 | 2.5 |
| 2022-11-01 | 0 |
| 2022-12-01 | 5.08475 |
| 2023-01-01 | 0 |

**Months**

**Figure 2** Monthly occurrence patterns of *Campylobacter* spp. in the exposure environments of diarrheic children at the study area (November 2021 to January 2023).

The contamination rate of food with *Campylobacter* spp. was 4.2%, with 3.2% in meat (3.2%, OR=0.59; 95% CI: 0.12–3.02), 5.3% in milk (5.3%, OR=0.98; 95% CI: 0.10–10.04), and 5.4% in other food categories (**Table 3**; **Table 4**). However, there were no significant differences in the distribution of *Campylobacter* spp. among foods prepared at households, those associated with marketplaces, and food samples collected from slaughterhouses. The logistic regression analysis demonstrated that the likelihood of *Campylobacter* spp. contamination in the environment was 5.8 times higher in the presence of poultry feces (17.7%; OR = 5.8; CI: 1.1–30.6) compared to shoat feces. The occurrence of *Campylobacter* spp. in animal wastewater was 5.4%, whereas its correspondence in mixed waste from both animals and humans was 13.8% (**Table 4**).

**Table 4:** Descriptive statistics of *Campylobacter* spp. occurrence by tracked specific animal species, food and environmental sample types and sources in eastern Ethiopia

| Sample type and source | | Negative | Positive | OR | 95% CI | P value |
| --- | --- | --- | --- | --- | --- | --- |
| Animal feces | Cattle and Camel | 71(95.95) | 3(4.05) | 1.14 | 0.18–7.07 | 0.89 |
|  | poultry | 28(82.35) | 6(17.65) | 5.79 | 1.10–30.56 | 0.04 |
|  | Shoat | 54(96.43) | 2(3.57) | Ref | ref | – |
|  | From abattoirs | 35(94.59) | 2(5.41) | 0.73 | 0.14–3.67 | 0.7 |
|  | From households | 29(93.55) | 2(6.45) | 0.88 | 0.17–4.46 | 0.87 |
|  | From marketplaces | 89(92.71) | 7(7.29) | Ref |  |  |
| Food | Meat | 90(96.77) | 3(3.23) | 0.59 | 0.12–3.02 | 0.53 |
|  | Milk | 18(94.74) | 1(5.26) | 0.98 | 0.10–10.04 | 0.98 |
|  | Other food | 53(94.64) | 3(5.36) | Ref | Ref | – |
|  | Abattoir-linked | 51(98.08) | 1(1.92) | 0.76 | 0.07–8.54 | 0.82 |
|  | Household-linked | 33(89.19) | 4(10.81) | 4.67 | 0.81–26.74 | 0.08 |
|  | Market-linked | 77(97.47) | 2(2.53) | Ref | – | – |
| Waste-water | From animals | 35(94.6) | 2(5.41) | 0.36 | 0.06–2.11 | 0.26 |
|  | Mixed waste | 25(86.21) | 4(13.79) | Ref | Ref | – |

Mixed waste:- mixed waste-water from both humans and animals

**Discussions**

*Campylobacter* spp. pose a significant health risk and are commonly acquired through consuming contaminated food, coming into contact with animals, and being exposed to environmental samples. In the present study, *Campylobacter* spp. were isolated from all sample types, with the exception of water sources. The overall *Campylobacter* occurrence rate of 5.5% in the physical exposure of diarrheic children in this study is comparable with the PCR results of a study conducted in Egypt (26). However, this prevalence is lower than a recent report from Ethiopia (27), which documented a prevalence of 7.7% in different environmental samples. Moreover, a pooled prevalence rate of 10.2% (10) and 14.9% (28) *Campylobacter* spp. in the environment was reported in a meta-analysis study conducted in Ethiopia and Iran, respectively.

The current study identified only *C. jejuni* and *C. coli,* with *C. jejuni* generally being a more common *Campylobacter* spp. Likewise, a recent study reported exclusively these two species, with *C. jejuni* as the predominant species in the environment in Ethiopia (27). Another study also documented a similar scenario, but it also reported the presence of *C. fetus* (13). *Campylobacter* spp. are fastidious microaerophile pathogens, but some species, like *C. jejuni*, exhibit aerotolerance and starvation survival that could be attributed to their high prevalence in the environment (29). On the other hand, the disparity in the occurrence of *Campylobacter,* like other enteric pathogens, among studies may be associated with differences in epidemiological risk factors (30,31).

Moreover, the occurrence of *Campylobacter* showed no significant difference (P > 0.05) between Harar town and Kersa district, highlighting its importance in both urban and rural settings of the current study area. Similarly, a study conducted in Ethiopia found no statistical variances among study sites (27). Other studies have also described that diarrheagenic pathogens remain a significant concern in resource-limited countries like Ethiopia (8,32), indicating poor environmental sanitation regardless of the setting. However, the health impacts of foodborne pathogens, including *Campylobacter*, is further aggravated in rural areas (14).

The occurrence of Campylobacter spp. exhibited fluctuations in monthly patterns throughout the study period, spanning from November 2021 to January 2023, with a peak in February 2022 during the winter season and the second highest occurrence observed during the rainy season. Likewise, it has been described that the occurrence of some diarrheagenic FBPs is seasonal, peaking at different times of the year (33). A previous study has also reported that Campylobacter occurrence peaks during the summer and has a smaller winter peak in many European countries, like Austria, Belgium, Finland, Germany, Luxembourg, the Netherlands, Switzerland, and Sweden (34). A recent study conducted in Ethiopia also reported an 11% occurrence rate of Campylobacter in milk during the dry season (7) and 20% during the wet season (35). The highest occurrence of Campylobacter during the winter season in our study could be linked to water scarcity for proper food sanitation and personal hygiene practices during the dry season, as previously described (36). In many villages in and around our study area, it is common to use shared water sources (water points) for various purposes, including animal drinking, irrigation, food preparation, and other purposes during the dry season.

Food samples accounted for 4.2% of the occurrence of *Campylobacter* spp. in the environment (4.17%, OR = 0.44; 95% CI: 0.14–1.35). However, (10) reported 9.3% *Campylobacter* spp. prevalence in food in Ethiopia. The absence of notable statistical variations in the distribution of *Campylobacter* among meat, milk, and other food categories within the study area demonstrates that the risk of contracting *Campylobacter*iosis remains consistent regardless of the type of food consumed. The relatively higher rate of *Campylobacter* in food samples collected from households may be attributed to contamination with poultry feces (37), as there were typically more poultry present at home than other sites in this study area.

The study found that the prevalence of *Campylobacter* in meat was lower compared to rates recently reported in meat from Ethiopia, 6.8% (27), in beef offals from Queensland, 10%, and from New South Wales, 21% (38), and in goat meat in Africa, 29.7% (39). Data from many European countries have shown a 12.9% occurrence rate of *Campylobacter* in ready-to-eat meat and meat products and 79.7% in non-ready-to-eat meat and meat products (34). The majority of meat samples analysed in this study were collected at the abattoir level or upon their arrival at butcher shops or households. Hence, the comparable occurrence rate of *Campylobacter* spp. in meat and in samples collected from abattoirs may indicate a potential link between slaughterhouse waste and *Campylobacter* contamination of meat.

Among the tracked milk samples, 5.26% tested positive for Campylobacter. This finding is comparable to the prevalence rate of *Campylobacter* (5.4%) that has been documented in non-ready-to-eat milk and milk products (34). However, previous studies conducted in Ethiopia have reported higher rates of *Campylobacter* in milk, at rates of 11% (7) and 20% (35). Furthermore, a study analysing data from many European countries has reported a 24.2% occurrence of *Campylobacter* in ready-to-eat milk and milk products (34). As the consumption of raw milk/meat and meat/milk products is common in Ethiopia, our study points out the need for the identification of potential sources of contamination to implement appropriate measures ensuring the safety and quality of meat and milk products, thereby safeguarding the public from further infections. It is also crucial to educate the public about the risks associated with consuming raw meat and milk.

Moreover, *Campylobacter* was detected in other food items—RTE foods, fruits, vegetables, juices, and leftover injera mixed with wot—at a rate of 5.4%. In contrast, a lower occurrence rate of 0.3% for *Campylobacter* was reported in juices and other non-alcoholic beverages from Ethiopia (40). Apart from Ethiopia, a meta-analysis study has reported 0.53% *Campylobacter* occurrence rates in vegetables, fruits, and fresh produce (41), while 3.3% in non-ready-to-eat fruit, vegetables, and juices has also been demonstrated in a previous study (34). Hence, our finding indicates that the hygienic practices in relation to such food items are inadequate and require attention. It is also important to note that contamination of fresh fruits and vegetables with Campylobacter can occur through various means, including contact with contaminated water, soil, or during handling and transportation. However, the contamination rate of ready-to-eat foods like fruit, vegetables, and juices with *Campylobacter* was documented at a significantly higher rate of 36.6%, making them the primary contributor to human *Campylobacter*iosis (34).

The carriage rate of *Campylobacter* in wastewater, at 9.1%, was found to be higher than its correspondence in food or animal feces. This higher prevalence in wastewater is likely attributed to the presence of a mixture of human and animal feces, particularly poultry feces, in the wastewater, leading to an increased occurrence rate. However, a much higher prevalence of 52.97% for *Campylobacter* (42) has been documented in wastewater. Furthermore, another study has highlighted that wastewater serves as an ideal reservoir for pathogens and acts as a common medium for their dissemination (43). Consequently, the utilization of wastewater as irrigation water poses a potential risk of contaminating agricultural produce, particularly vegetables on farmlands. In Sub-Saharan Africa, a significant number of vegetable crops are irrigated mainly using wastewater, giving an opportunity for some microbes to contaminate the plants and subsequently affect consumers (44).

In general, this study took place between November 2021 (early post-COVID) and January 2023, and the hygienic measures experienced during the COVID-19 pandemic may have been attributed to the lowered occurrence of *Campylobacter* in food samples. Nevertheless, discrepancies in food types as well as non-uniform protocols for sampling and identification could have resulted in heterogeneity among studies, although some high prevalence findings could be factual due to an extensive variety of raw and RTE foods (45).

As shown in **Table 4**, *Campylobacter* was detected in animal feces collected from abattoirs, households, and marketplaces, with no statistical differences (p ≤ 0.05) in its occurrence across sample sources. However, a significant association between *Campylobacter* occurrence and animal feces collected from households has previously been reported (13). The overall prevalence of *Campylobacter* in animals in this study is lower than the findings of previous studies conducted by (12) and (10), who, respectively, reported 56.5% and 14.6%.

Moreover, a higher prevalence of 13.5% at the farm level and 25.3% at the abattoir level has been documented in domestic animal feces. Additionally, 31.1% at the farm level, 37.7% at marketplaces, and 61.9% at the abattoir level were previously reported in poultry (28). The variation in the prevalence rates may be due to differences in sample size and sampling protocol. In our case, the pooling of samples from the same species at the same site or in the same households may affect the prevalence rates. This lower prevalence could also be attributed to a higher likelihood of animals being exposed to the effects of antibiotics, as many animal owners in our study area administer modern antibiotic drugs and traditional medicine to their animals without a prescription. Furthermore, antibiotics are frequently used for purposes other than their intended use.

On the other hand, the occurrence of *Campylobacter* in poultry was found to be statistically higher (17.65%; P = 0.04) compared to cattle and camels, and shoat feces. It is widely acknowledged that food production animals are often identified as the main source of Campylobacter infections, with birds being recognized as the primary reservoirs, which may be due to their elevated body temperature (46). Similarly, a study conducted in Ethiopia reported a higher prevalence of *Campylobacter* spp. in poultry (86.6%) than in cattle (48%), sheep (39%), and goats (33.3%) feces (12)*.* Conversely, another study has documented that *Campylobacter* is a more prevalent pathogen in cattle (18.5%) and sheep (13.3%) than in poultry feces (13%) (13). Comparable to our study results, a study conducted in Iran reported a prevalence of 5.3% in cattle and 4% in camel feces (47)*.* The inclusion of a varying number of animal species in this study might also affect the prevalence of *Campylobacter.*

Interestingly, *Campylobacter* was not detected in the drinking water, recreation water, or irrigation water considered as water sources in this study. In contrast, a previous study (48) identified *Campylobacter* in different water sources. Specifically, a prevalence of 10% in drinking water from Ethiopia (13) and 52% in groundwater in Nigeria (49) have been documented for *Campylobacter* spp. The detection of *Campylobacter* or other Enterobacteriaceae in water is an indication of unsanitary fecal contamination of water bodies, which increases the risk of spreading pathogens. As a result, drinking untreated water from any water source poses a risk factor for acquiring water-borne pathogens, especially in developing regions (6,50). However, the implementation of improved hygienic measures and the lessons learned from the COVID-19 era in the community may lead to an enhancement in water contamination in Ethiopia. Nevertheless, it is important to note that this study, which had a limited sample size of only 40 water samples, cannot be generalized to conclude that water sources are free of *Campylobacter* contamination in eastern Ethiopia post-COVID-19. Additionally, the scope of this study was restricted to analysing water sources directly or indirectly used by families of cases identified during the study period.

**Conclusion and Recommendations**

*Campylobacter* is a zoonotic pathogen and poses a significant public health hazard, especially in low-income nations. This study identified *Campylobacter* in different samples, including human, animal, food, and environmental samples, at a prevalence rate of 6.5%. Interestingly, *Campylobacter* was detected in all sample sources and types, except for water sources, with no significant difference in its occurrence between rural and urban settings in Ethiopia (P≤ 0.05), demonstrating the important risks of contracting the pathogen when exposed to various samples regardless of the setting, especially during the dry season. Our findings also revealed that chicken droppings provide a higher risk of contamination of the environment than that of other animals. Therefore, the study suggests the need for one health approach to be implemented to reduce the risk of campylobacteriosis in both urban and rural areas. It specifically highlights the necessity of ensuring food safety and better wastewater management, including proper handling of animal excreta, particularly poultry droppings. Further studies employing large sample sizes and advanced diagnostic techniques, like whole genome sequencing and meta genomic analysis, are recommended to determine the relative contribution of each source to the occurrence of *Campylobacter*-associated diarrheal disease in children.

**Ethics approval and consent to participate**

The project had been granted ethical approval from the Institutional Health Research Ethics Review Committee (IHRERC) of Haramaya University, the National Research Ethics Committee, Ethiopia, and the Ministry of Education (Ref No: MoSHE/RD/14.1/9849/20).

**Authors’ contributions**

In general, the authors collectively contributed to design the study methodology, proposal writing, training, and supervision of data collectors. Additionally, TG and TH were responsible for funding acquisition, while DB conducted laboratory analysis, data entry, analysis, and interpretation of the results.

**Funding**

The study is part of FOCAL (Foodborne disease epidemiology, surveillance, and control in African LMIC). FOCAL is a multi-partner, multi-country research project co-funded by the Bill & Melinda Gates Foundation and the Foreign, Commonwealth, and Development Office (FCDO) of the United Kingdom Government [grant agreement investment ID OPP1195617]. However, the funders did not have the decision to publish or prepare the manuscript.

**Acknowledgements**

The authors would like to thank FOCAL research project funders and the Haramaya University School of Medical Laboratory for their cooperation in conducting the activities in their laboratory. We are also grateful to the FOCAL research project data collection and laboratory analysis teams.

**Availability of data**

The data used in this study are presented in the document, and complete data, including supplementary files (if any), may be accessible through the corresponding author.

**Competing interests**: All authors read and approved the final manuscript. Its contents are solely the responsibility of the authors. All authors declare that they have no competing interests.

**References**

1.	WHO. Campylobacter [Internet]. 2020 [cited 2023 Oct 22]. Available from: https://www.who.int/news-room/fact-sheets/detail/campylobacter

2.	Tobolowsky F, Laughlin M, R A, D P. Campylobacteriosis | CDC Yellow Book 2024.Travel-Associated Infections & Diseases [Internet]. 2023 [cited 2023 Oct 11]. Available from: https://wwwnc.cdc.gov/travel/yellowbook/2024/infections-diseases/campylobacteriosis

3.	Behailu Y, Hussen S, Alemayehu T, Assegu D, Id F. Prevalence, determinants, and antimicrobial susceptibility patterns of Campylobacter infection among under-five children with diarrhea at Governmental Hospitals in Hawassa city, Sidama, Ethiopia. A cross-sectional study. PLoS One [Internet]. 2022 [cited 2023 Oct 4];17(5):e0266976. Available from: https://doi.org/10.1371/journal.pone.0266976

4.	Paintsil EK, Masanta WO, Dreyer A, Ushanov L, Smith SI, Frickmann H, et al. Campylobacter in Africa – A specific viewpoint. Eur J Microbiol Immunol (Bp) [Internet]. 2023 Dec 21 [cited 2024 Apr 22];13(4):107. Available from: /pmc/articles/PMC10755667/

5.	Abay KA, Desalegn G, Weldu Y, Gebrehiwot GT, Gebreyohannes G, Welekidan LN, et al. Prevalence and Antimicrobial Resistance of Campylobacter Species and Associated Factors Among Under-Five Children with Diarrhea at Randomly Selected Public Health Facilities in Mekelle, Tigray, Ethiopia. Infect Drug Resist [Internet]. 2024 [cited 2024 Apr 22];17:495. Available from: /pmc/articles/PMC10860571/

6.	Belina D, Hailu Y, Gobena T, Hald T, Njage PMK. Prevalence and epidemiological distribution of selected foodborne pathogens in human and different environmental samples in Ethiopia: a systematic review and meta-analysis. One Heal Outlook [Internet]. 2021 Dec 3 [cited 2023 Sep 29];3(1):1–30. Available from: https://onehealthoutlook.biomedcentral.com/articles/10.1186/s42522-021-00048-5

7.	Admasie A, Eshetu A, Tessema TS, Vipham J, Kovac J, Zewdu A. Prevalence of Campylobacter species and associated risk factors for contamination of dairy products collected in a dry season from major milk sheds in Ethiopia. Food Microbiol. 2023 Feb 1;109:104145.

8.	Beyene AM, Gezachew M, Mengesha D, Yousef A, Gelaw B. Prevalence and drug resistance patterns of Gram-negative enteric bacterial pathogens from diarrheic patients in Ethiopia: A systematic review and meta-analysis. PLoS One [Internet]. 2022 Mar 1 [cited 2023 Sep 3];17(3):e0265271. Available from: https://journals.plos.org/plosone/article?id=10.1371/journal.pone.0265271

9.	Terefe Y, Deblais L, Ghanem M, Helmy YA, Mummed B, Chen D, et al. Co-occurrence of Campylobacter Species in Children From Eastern Ethiopia, and Their Association With Environmental Enteric Dysfunction, Diarrhea, and Host Microbiome. Front public Heal [Internet]. 2020 Apr 15 [cited 2023 Oct 12];8. Available from: https://pubmed.ncbi.nlm.nih.gov/32351922/

10.	Zenebe T, Zegeye N, Eguale T. Prevalence of Campylobacter species in human, animal and food of animal origin and their antimicrobial susceptibility in Ethiopia: a systematic review and meta-analysis. Ann Clin Microbiol Antimicrob [Internet]. 2020 Dec 1 [cited 2023 Oct 4];19(1):1–11. Available from: https://ann-clinmicrob.biomedcentral.com/articles/10.1186/s12941-020-00405-8

11.	Berhanu L, Bedru H, Gume B, Tolosa T, Kassa T, Getaneh A, et al. <p>Occurrence, Risk Factors, and Antimicrobial Susceptibility Test of Thermophilic <em>Campylobacter</em> Species of Bovine Carcass at Municipal Abattoir and Butcher Shops of Jimma Town, Southwest Ethiopia</p>. Infect Drug Resist [Internet]. 2021 Sep 15 [cited 2024 Mar 31];14:3753–62. Available from: https://www.dovepress.com/occurrence-risk-factors-and-antimicrobial-susceptibility-test-of-therm-peer-reviewed-fulltext-article-IDR

12.	Abamecha A, Assebe G, Tafa B, Wondafrash B. Prevalence of Thermophilic Campylobacter and their Antimicrobial Resistance Profile in Food Animals in Lare District, Nuer Zone, Gambella, Ethiopia. J Drug Res Dev. 2016;1(2).

13.	Chala G, Eguale T, Abunna F, Asrat D, Stringer A. Identification and Characterization of Campylobacter Species in Livestock, Humans, and Water in Livestock Owning Households of Peri-urban Addis Ababa, Ethiopia: A One Health Approach. Front Public Heal [Internet]. 2021 Dec 2 [cited 2023 Oct 5];9:750551. Available from: /pmc/articles/PMC8677049/

14.	Asfaw T, Genetu D, Shenkute D, Shenkutie TT, Amare YE, Yitayew B. Foodborne Pathogens and Antimicrobial Resistance in Ethiopia: An Urgent Call for Action on “One Health.” Infect Drug Resist. 2022;15(August):5265–74.

15.	Seyoum A, Urgessa K, Gobena T. Risk Management and Healthcare Policy Dovepress Community knowledge and the role of health extension workers on integrated diseases among households in East Hararghe Zone, Ethiopia. 2016 [cited 2023 Sep 3]; Available from: http://dx.doi.org/10.2147/RMHP.S105608

16.	UNICEF. Situation Analysis of Children and Women: Harari. In 2019 [cited 2023 Sep 3]. Available from: https://www.unicef.org/ethiopia/media/2546/file/Harari region .pdf

17.	ERCS. Ethiopia Harar Town Integrated WASH Response Skybird Project | Skybird Programme. Ethiopian Red Cross Society (ERCS) [Internet]. 2022 [cited 2023 Sep 25]. Available from: https://skybird-wash.net/2022/11/27/1668/

18.	Belina D, Gobena T, Kebede A, Chimdessa M, Hailu Y, Hald T. Occurrence of Diarrheagenic Pathogens and Their Coinfection Profiles in Diarrheic Under Five Children and Tracked Human Contacts in Urban and Rural Settings of Eastern Ethiopia. Microbiol Insights [Internet]. 2023 Jan [cited 2023 Nov 1];16. Available from: /pmc/articles/PMC10510352/

19.	Tm B, I A, M IW, Ferro I, D II. Evaluation of the Direct and Enrichment Iso 10272 Methods for the Detection of Campylobacter Spp. in Chicken Meat. Brazilian J Poult Sci [Internet]. 2016 [cited 2024 Apr 28];18(1):023–8. Available from: http://dx.doi.org/10.1590/1516-635x1801023-028

20.	Huang H, Garcia MM, Huang H, Garcia MM. Isolation and Identification of <em>Campylobacter</em> spp. from Food and Food-Related Environment. Campylobacter [Internet]. 2022 Sep 7 [cited 2024 Apr 28]; Available from: https://www.intechopen.com/chapters/81596

21.	Audu BJ, Norval S, Bruno L, Meenakshi R, Marion M, Forbes KJ. Genomic diversity and antimicrobial resistance of Campylobacter spp. from humans and livestock in Nigeria. J Biomed Sci [Internet]. 2022 Dec 1 [cited 2023 Nov 4];29(1):7. Available from: /pmc/articles/PMC8788075/

22.	Vondrakova L, Pazlarova J, Demnerova K. Detection, identification and quantification of Campylobacter jejuni, coli and lari in food matrices all at once using multiplex qPCR. Gut Pathog [Internet]. 2014 May 9 [cited 2024 May 5];6(1):1–9. Available from: https://gutpathogens.biomedcentral.com/articles/10.1186/1757-4749-6-12

23.	Platts-Mills JA, Liu J, Gratz J, Mduma E, Amour C, Swai N, et al. Detection of Campylobacter in Stool and Determination of Significance by Culture, Enzyme Immunoassay, and PCR in Developing Countries. J Clin Microbiol [Internet]. 2014 [cited 2024 May 5];52(4):1074. Available from: /pmc/articles/PMC3993515/

24.	Zymo_Research. ZymoScript^TM^ One-Step RT-qPCR Kit Simple and sensitive qPCR detection of RNA targets Product Information.

25.	Zymo Research Kit. Quick-DNA^TM^ Fungal/Bacterial Miniprep Kit [Internet]. 2021 [cited 2024 Mar 10]. Available from: www.zymoresearch.com

26.	Karmi M. PREVALENCE OF CAMPYLOBACTER SPP. AND ITS PATHOGENIC GENES IN POULTRY MEAT, HUMAN AND ENVIRONMENT IN ASWAN, UPPER EGYPT. Assiut Vet Med J [Internet]. 2019 Apr 9 [cited 2024 Apr 29];65(161):151–8. Available from: https://avmj.journals.ekb.eg/article_168777.html

27.	Yirgalem M, Kemal J, Bekele M. Isolation, molecular detection and antimicrobial susceptibility proles of Campylobacter from camel meat, feces and processing environments at municipal abattoirs in eastern Ethiopia. 2024 [cited 2024 Apr 29]; Available from: https://doi.org/10.21203/rs.3.rs-4173994/v1

28.	Ansarifar E, Riahi SM, Tasara T, Sadighara P, Zeinali T. Campylobacter prevalence from food, animals, human and environmental samples in Iran: a systematic review and meta-analysis. BMC Microbiol [Internet]. 2023 Dec 1 [cited 2024 Apr 29];23(1). Available from: /pmc/articles/PMC10170724/

29.	Bronowski C, James CE, Winstanley C. Role of environmental survival in transmission of Campylobacter jejuni. FEMS Microbiol Lett [Internet]. 2014 [cited 2024 Apr 29];356(1):8–19. Available from: https://pubmed.ncbi.nlm.nih.gov/24888326/

30.	Bagamian KH, Anderson JD, Muhib F, Cumming O, Laytner LA, Wierzba TF, et al. Heterogeneity in enterotoxigenic Escherichia coli and shigella infections in children under 5 years of age from 11 African countries: a subnational approach quantifying risk, mortality, morbidity, and stunting. Lancet Glob Heal [Internet]. 2020;8(1):e101–12. Available from: http://dx.doi.org/10.1016/S2214-109X(19)30456-5

31.	Reiner RC, Graetz N, Casey DC, Troeger C, Garcia GM, Mosser JF, et al. Variation in Childhood Diarrheal Morbidity and Mortality in Africa, 2000–2015. N Engl J Med [Internet]. 2018 Sep 20 [cited 2023 Oct 16];379(12):1128–38. Available from: https://www.cidrap.umn.edu/public-health/studies-diarrheal-disease-rates-vary-across-africa-world

32.	Abera K, Anticho TL, Ali MM. Salmonella and Shigella and antimicrobial susceptibility profiles among adult patients with complaints of diarrhea at Hawassa comprehensive specialized hospital, Hawassa, Ethiopia . SAGE Open Med. 2021 Jan;9:205031212110009.

33.	Platts-Mills JA, Babji S, Bodhidatta L, Gratz J, Haque R, Havt A, et al. Pathogen-specific burdens of community diarrhoea in developing countries: a multisite birth cohort study (MAL-ED). Lancet Glob Heal [Internet]. 2015 [cited 2023 Sep 3];3(9):e564–75. Available from: https://pubmed.ncbi.nlm.nih.gov/26202075/

34.	Liu F, Lee SA, Xue J, Riordan SM, Zhang L. Global epidemiology of campylobacteriosis and the impact of COVID-19. Front Cell Infect Microbiol. 2022 Nov 28;12:979055.

35.	Admasie A, Eshetu A, Sisay T, Vipham J, Kovac J, Zewdu A. Seasonal Variation in the Prevalence and Antimicrobial Resistance of Campylobacter in Milk and Milk Products in Ethiopia. Int Dairy J [Internet]. 2023 [cited 2024 Mar 31];149. Available from: https://papers.ssrn.com/abstract=4479841

36.	Kimemia JM, Kariuki JG, Odongo AO, Murima PNAA. Factors Associated with Foodborne Pathogens among Food Handlers: A Case Study of Thika, Kiambu County, Kenya. Adv Public Heal. 2023;2023.

37.	Perez-Arnedo I, Gonzalez-Fandos E. Prevalence of Campylobacter spp. in Poultry in Three Spanish Farms, A Slaughterhouse and A Further Processing Plant. Foods 2019, Vol 8, Page 111 [Internet]. 2019 Mar 26 [cited 2024 Apr 6];8(3):111. Available from: https://www.mdpi.com/2304-8158/8/3/111/htm

38.	Walker LJ, Wallace RL, Smith JJ, Graham T, Saputra T, Symes S, et al. Prevalence of Campylobacter coli and Campylobacter jejuni in Retail Chicken, Beef, Lamb, and Pork Products in Three Australian States. J Food Prot [Internet]. 2019 [cited 2024 Apr 1];82(12):2126–34. Available from: https://pubmed.ncbi.nlm.nih.gov/31729918/

39.	Thomas KM, de Glanville WA, Barker GC, Benschop J, Buza JJ, Cleaveland S, et al. Prevalence of Campylobacter and Salmonella in African food animals and meat: A systematic review and meta-analysis. Int J Food Microbiol [Internet]. 2020 Feb 2 [cited 2023 Oct 16];315. Available from: /pmc/articles/PMC6985902/

40.	Hiko A, Muktar Y. Levels of microbial contamination in non-alcoholic beverages from selected eastern Ethiopian towns markets. SciAf [Internet]. 2020 Mar 1 [cited 2024 Apr 1];7:e00223. Available from: https://ui.adsabs.harvard.edu/abs/2020SciAf...700223H/abstract

41.	Mohammadpour H, Berizi E, Hosseinzadeh S, Majlesi M, Zare M. The prevalence of Campylobacter spp. in vegetables, fruits, and fresh produce: a systematic review and meta-analysis. Gut Pathog [Internet]. 2018 Sep 27 [cited 2024 May 2];10(1). Available from: /pmc/articles/PMC6158901/

42.	Zhang S, Shi J, Li X, Tiwari A, Gao S, Zhou X, et al. Wastewater-based epidemiology of Campylobacter spp.: A systematic review and meta-analysis of influent, effluent, and removal of wastewater treatment plants. Sci Total Environ [Internet]. 2023 Dec 10 [cited 2024 Apr 5];903. Available from: https://pubmed.ncbi.nlm.nih.gov/37597560/

43.	Boulani A El, Mimouni R, Mannas H, Fatima, Hamadi, Chaouqy N, et al. Salmonella in Wastewater: Identification, Antibiotic Resistance and the Impact on the Marine Environment. Curr Top Salmonella Salmonellosis [Internet]. 2017 Apr 5 [cited 2023 Sep 12]; Available from: https://www.intechopen.com/chapters/54428

44.	Balali GI, Yar DD, Afua Dela VG, Adjei-Kusi P. Microbial Contamination, an Increasing Threat to the Consumption of Fresh Fruits and Vegetables in Today’s World. Int J Microbiol. 2020;2020.

45.	Paudyal N, Anihouvi V, Hounhouigan J, Matsheka MI, Sekwati-Monang B, Amoa-Awua W, et al. Prevalence of foodborne pathogens in food from selected African countries - A meta-analysis. Int J Food Microbiol [Internet]. 2017 May 16 [cited 2023 Sep 9];249:35–43. Available from: https://pubmed.ncbi.nlm.nih.gov/28271855/

46.	Korsak D, Maćkiw E, Rozynek E, Zyłowska M. Prevalence of Campylobacter spp. in Retail Chicken, Turkey, Pork, and Beef Meat in Poland between 2009 and 2013. J Food Prot [Internet]. 2015 May 1 [cited 2024 Apr 30];78(5):1024–8. Available from: https://pubmed.ncbi.nlm.nih.gov/25951401/

47.	Rahimi E, Alipoor-Amroabadi M, Khamesipour F. Investigation of prevalence of thermotolerant Campylobacter spp. in livestock feces. https://doi.org/101139/cjas-2015-0166 [Internet]. 2017 Mar 10 [cited 2024 May 3];97(2):207–13. Available from: https://bioone.org/journals/canadian-journal-of-animal-science/volume-97/issue-2/cjas-2015-0166/Investigation-of-prevalence-of-thermotolerant-Campylobacter-spp-in-livestock-feces/10.1139/cjas-2015-0166.full

48.	Kemp R, Leatherbarrow AJH, Williams NJ, Hart CA, Clough HE, Turner J, et al. Prevalence and Genetic Diversity of Campylobacter spp. in Environmental Water Samples from a 100-Square-Kilometer Predominantly Dairy Farming Area. Appl Environ Microbiol [Internet]. 2005 Apr [cited 2024 May 2];71(4):1876. Available from: /pmc/articles/PMC1082521/

49.	Ugboma AN, Salihu MD, Magaji AA, Abubakar MB. Prevalence of campylobacter species in ground water in Sokoto, Sokoto state, Nigeria. Vet World. 2013 Jun;6(6):285–7.

50.	Yasin M, Ketema T, Bacha K. Physico-chemical and bacteriological quality of drinking water of different sources, Jimma zone, Southwest Ethiopia. BMC Res Notes. 2015;8:541.

**Figure 3.** Characteristic *Campylobacter* colony and the spectrophotometric readings for DNA purity obtained using NanoPhotometer-Mettler-Toledo, depicted in a pictorial presentation.

|  |  | |
| --- | --- | --- |
| A. Characteristic *Campylobacter* pure colonies on mCCDA | | |
|  | |  |
|  | |  |
| B. The spectrophotometric readings for DNA purity obtained from some samples | | |
