# Supplementary material for: Occurrence and diversity of Campylobacter species in diarrheic children and their exposure environments in Ethiopia
Source: PLOS Glob Public Health. 2024 Oct 29;4(10):e0003885. doi: 10.1371/journal.pgph.0003885 (PMC11521251; doi:10.1371/journal.pgph.0003885)
Supplement: Attachment — Submitted filename: Comments for the PGPH-D-24-01065_AAM.docx [file pgph.0003885.s002.docx]

**General comments**

The work is presented in a journal style, the methodology is clear, straightforward and acceptable for this kind of a work, Paper should be accepted with some minor corrections.

General comments are to avoid personifications in the writing, too long sentences and check for some few grammatical errors in the paper.

**Abstract**

Maintain using single decimal or no decimal place (see line 21)

No results from the genomic and bioinformatics parts of the method

**Introduction**

Looks like the study is done on animals, food and environmental samples in immediate diarrheic environment of UFC and that is not captured in the tittle.

Need to be clearer in the problem statement and justification, develop a clear foundation as to why you had to do the study.

Re-construct the objective to be simple and clear to the reader

**Methods**

Based on health indicators, what was the cue to go to into that specific study site? This information on is not captured

Be aware of the use of the word tracing and tracking, in a context of the paper its more of tracing rather than tracking.

How was whole genome sequencing done, what method was used, was it sanger, next generation? No enough details.

Detailed explanation of bioinformatics analysis is missing

**Results**

There is a consistent omission of the whole genome species identification data, what was the output of sequence data? similarity index of the species to the Reference in GeneBank? No any plot from sequencing?

Consistence with the use of decimal places, choose to either use one or two or no decimal places in proportions presentations,

**Discussion**

The discussion is well written, it covers all the important parts of the results, however, there has to be a link between the identification used and its influence on the results of identification.
