# Supplementary material for: Occurrence and diversity of Campylobacter species in diarrheic children and their exposure environments in Ethiopia
Source: PLOS Glob Public Health. 2024 Oct 29;4(10):e0003885. doi: 10.1371/journal.pgph.0003885 (PMC11521251; doi:10.1371/journal.pgph.0003885)
Supplement: Attachment — Submitted filename: Authors Response_PGPH-D-24-01096.docx [file pgph.0003885.s003.docx]

**To PLOS Global Public Health Academic Manager(s)**

**Subject: A rebuttal letter**

Dear Editor(s),

We would like to thank the PGPH editor(s) and reviewers for their constructive comments and suggestions on our manuscript, PGPH-D-24-01096, **“Detection and Occurrence Patterns of Campylobacter species in the Exposure Environments of Diarrheic Children in Ethiopia,**". Based on reviewers’ comments (particularly reviewer #1), we have now incorporated whole-genome sequencing (WGS)-based phylogenetic tree analysis, and the title of the manuscript has been modified. As a result, two additional co-authors who made significant contributions to the genomic aspects have been included in the author list. Language editing and other revisions, indicated with track changes in the older version of the manuscript, have also been made to the manuscript. Additionally, the authors addressed the specific comments and clarity questions raised by the editor(s) and reviewers, as described below.

We are now submitting the revised version of the manuscript and look forward to hearing feedback from the journal.

Thank you

The manuscript authors

A. **Authors Response to Editor(s)**
1. In the ethics statement in the Methods, you have specified that verbal consent was obtained. Please provide additional details regarding how this consent was documented and witnessed, and state whether this was approved by the IRB.

― This manuscript was produced as a part of the FOCAL project, which received ethical approval for its entire research protocol, including the procedures for obtaining consent and assent from participants. The study collected two distinct sample types: children's stool samples, or "diarrhea," and traced contact samples, or "exposure sources." Thus, after the overall project protocol was ethically approved, the researchers obtained cooperation from the community in Harar town and Kersa district by officially informing the relevant local authorities and health bureaus, who then communicated with the community to cooperate in the provision of samples and related data.

― In the old version of the manuscript, we stated that verbal consent was obtained, indicating that caretakers did not provide separate signatures for the project. Rather, they were informed of the objectives of the study and consented to the collection of children's stool (diarrhea) samples, which were collected at healthcare facilities in collaboration with local health professionals, as the children sought routine stool tests for treatment. This statement has been modified in the revised version of the manuscript.

2. Please amend your detailed Financial Disclosure statement. This is published with the article. It must therefore be completed in full sentences and contain the exact wording you wish to be published.

― The financial disclosure statement and role of the funders have been rephrased and corrected in the revised version of the manuscript.

3. We do not publish any copyright or trademark symbols that usually accompany proprietary names, eg (R), (C), or TM (e.g. next to drug or reagent names). Please remove all instances of trademark/copyright symbols throughout the text, including TM on page 11.

― All trademark symbols have been removed.

4. In the online submission form, you indicated that "The data used in this study are presented in the document, and complete data, including supplementary files (if any), may be accessible through the corresponding author."

― The summary data of study participants or households has been presented in the results section. However, tagging specific participants or household data would compromise the privacy of the participants. However, to address the data availability policy of PLOS, the raw sequence data used in this manuscript has now been made publicly available. Specifically, the whole-genome sequencing (WGS) data has been submitted to the European Nucleotide Archive (ENA) under the accession number PRJEB73590.

5. Please review your reference list to ensure that it is complete and correct. If you have cited papers that have been retracted, please include the rationale for doing so in the manuscript text, or remove these references and replace them with relevant current references. Any changes to the reference list should be mentioned in the rebuttal letter that accompanies your revised manuscript.

― The references have been thoroughly checked to ensure they are complete and correct according to the journal's guidelines. The references are now complete and correct as per the journal's requirements.

― Generally, no retracted papers have been cited in the manuscript. However, based on the data availability issue and the reviewers' comments regarding the whole-genome sequencing (WGS) data, a WGS-based phylogenetic tree analysis has now been incorporated into the manuscript. As a result, the introduction section has been revised, and new references have been cited to support the newly incorporated phylogenetic tree analysis results. Additionally, the incorporated phylogenetic tree analysis results were discussed, for which new references have been cited.

**Authors Response to Reviewer comments (**# **Reviewer1)**

We are grateful for the constructive comments and suggestions you provided for the improvement of our manuscript. The authors have checked all comments provided and have made corrections accordingly.

**General**

General comments are to avoid personifications in the writing, too long sentences and check for some few grammatical errors in the paper.

― The authors have been addressed grammatical errors with a professional language edition, and personifications in the writing and the use of too long sentences have been corrected in the revised version of the manuscript.

― Based on the data availability Policy and your (the reviewer # 2) comments, a whole-genome sequencing (WGS)-based phylogenetic tree analysis has now been incorporated into the manuscript. As a result, the introduction section has been revised to support the newly incorporated phylogenetic results. Additionally, the incorporated phylogenetic tree analysis results have been discussed separately.

**Specific responses are given point by point as per comments and suggestions**.

**Abstract**

1. Maintain using single decimal or no decimal place (see line 21)

― The comment has been accepted, and numerical values have been corrected to one decimal place throughout the manuscript.

2. No results from the genomic and bioinformatics parts of the method

― The authors have accepted this comment, and WGS-based phylogenetic tree analysis has now been incorporated into the manuscript. As a result, the abstract and introduction sections have been revised to support the newly incorporated phylogenetic results. Additionally, the incorporated phylogenetic tree analysis results have been discussed separately.

**Introduction**
1. Looks like the study is done on animals, food and environmental samples in immediate diarrheic environment of UFC and that is not captured in the tittle. Need to be clearer in the problem statement and justification, develop a clear foundation as to why you had to do the study.

―The introduction, including the problem statement and justification, has been revised based on the comments provided. However, in our view, incorporating terms like animals, food, and environmental samples in the title would make it overly lengthy. Instead, these can be collectively represented by the term "exposure sources." To provide clarity, the term exposure source," including "direct exposure sources" and "indirect exposure sources," has been defined and incorporated into the methodology under the "Sample Types and Collection Procedures" section.

3. Define, Food-borne Pathogens

― The comment has been accepted, and the term FBP is defined

4. Re-construct the objective to be simple and clear to the reader

― Considering the reviewer comments and to incorporate the newly added phylogenetic tree analysis results, the study objective has been modified. The revised objective now encompasses the expanded scope of the analysis.

**Methods**

1. Based on health indicators, what was the cue to go to into that specific study site? This information on is not captured

― The comment has been considered and described in the revised version of the manuscript.

2. Be aware of the use of the word tracing and tracking, in a context of the paper its more of tracing rather than tracking.

― Accepted, and throughout the manuscript, the word tracking has been replaced by tracing.

3. How was whole genome sequencing done, what method was used, was it sanger, next generation? No enough details. Detailed explanation of bioinformatics analysis is missing.

― In the previous version of the manuscript, a detailed explanation of the bioinformatics analysis was missing, as the whole-genome sequencing (WGS) data was only described in the context of species identification using the "SpeciesFinder" tool. This was due to the expectation of publishing a separate manuscript focused on the WGS-based phylogenetic tree analysis of *Campylobacter*. However, based on the reviewer comments, the detailed WGS data and bioinformatics analysis have now been incorporated into the current manuscript to enrich the overall content.

― Raw sequences have now been submitted to the European Nucleotide Archive (ENA) under the accession number PRJEB73590.

**Results**

1. There is a consistent omission of the whole genome species identification data, what was the output of sequence data? similarity index of the species to the Reference in GeneBank? No any plot from sequencing?

― This comment has been accepted, and the WGS output is incorporated into the revised manuscript.

― In the result part of the revised manuscript, table positions have been rearranged so that prevalence data (risk factors) are presented first and followed by the WSG analysis results.

― As per the journal guidelines, figures have been submitted separately.

2. Consistence with the use of decimal places, choose to either use one or two or no decimal places in proportions presentations,

― Has been considered and corrected uniformly throughout the manuscript.

**Discussion**

1. The discussion is well written, it covers all the important parts of the results, however, there has to be a link between the identification used and its influence on the results of identification.

― Has been considered and corrected as indicate by track changes in the revised manuscript.

**Authors Response to Reviewer comments (**# **Reviewer2)**

We are grateful for the constructive comments and suggestions you provided for the improvement of our manuscript. The authors checked all comments provided and have made corrections accordingly.

**Changes that has been made to the manuscript:**

― The authors have addressed the overall grammatical errors with a professional language edition.

― With the expectation of publishing a separate manuscript focused on the WGS-based phylogenetic tree analysis of *Campylobacter*, the phylogenetic tree analysis was missing from the previous version of the manuscript. However, based on the data availability policy of PLOS and comments from other reviewers of this manuscript, the phylogenetic tree analysis results have now been incorporated. As a result, the title and objective of the study have been modified accordingly.

― Raw sequences have now been submitted to the European Nucleotide Archive (ENA) under the accession number PRJEB73590.

 ― In the result part of the revised manuscript, table positions have been rearranged so that prevalence data (risk factors) are presented first and followed by the WSG analysis results.

― As per the journal guidelines, figures have been submitted separately.

― The suggestion to improve grammar and clarity of the statement (in line 324) is “However, (10) reported 9.3% Campylobacter spp. prevalence in food in Ethiopia’’. Has been accepted and corrected.

―The comment given to correct the description in the discussion section for the improvements in the grammar and clarity of the statement (lines 383 to 385): “The overall prevalence of *Campylobacter* in animals in this study is lower than the findings of previous studies conducted by (12) and (10), who, respectively, reported 56.5% and 14.6%." Has also been accepted and corrected.
