# Supplementary material for: Occurrence and diversity of Campylobacter species in diarrheic children and their exposure environments in Ethiopia
Source: PLOS Glob Public Health. 2024 Oct 29;4(10):e0003885. doi: 10.1371/journal.pgph.0003885 (PMC11521251; doi:10.1371/journal.pgph.0003885)
Supplement: Attachment — Submitted filename: PGPH-D-24-01096_Response to Reviewers.docx [file pgph.0003885.s004.docx]

**To PLOS Global Public Health Academic Manager(s)**

**Subject: Resubmission of revised manuscript**

Dear Editorial manager(s),

We would like to thank the PGPH editor(s) and reviewers for their constructive comments and suggestions on our manuscript, PGPH-D-24-01096, **“Occurrence and Diversity of *Campylobacter* species in Diarrheic Children and Their Exposure Environments in Ethiopia,**". Based on reviewers’ comments, we have made minor revision to the manuscript by incorporating the given comments. Language editing and other typographical errors correction were also made to the manuscript as indicated with track changes or highlights in the v2 version of the manuscript. Additionally, the authors addressed the specific comments and clarity questions raised by the editor(s) and reviewers, as described below. However, we found that some of the comments given by reviewer 2 and one suggestion provided by reviewer 3 was already addressed in the revised version of the manuscript (V1) submitted to the journal last month.

We are now submitting the revised version of the manuscript (V2) and look forward to hearing feedback from the journal.

Thank you

The manuscript authors

A. **Authors Response to Editor(s)**
1. In the ethics statement in the Methods, you have specified that verbal consent was obtained. Please provide additional details regarding how this consent was documented and witnessed, and state whether this was approved by the IRB.

― We have made some revision to the ethical approval statement.

― **Clarity:** This manuscript was produced as a part of the FOCAL project, which received ethical approval for its entire research protocol, including the procedures for obtaining consent/assent from participants/caretakers. The project analysed multiple diarrheagenic pathogens, although this particular manuscript focuses on *Campylobacter*. For instance, as part of this project, a work conducted on *Salmonella* using the same study protocol (sample collection) and ethical approval used for the same has been published. <https://pubmed.ncbi.nlm.nih.gov/39187763/>

Furthermore, here is the summary of the informed voluntary consent declaration form used in the study.

**Annex XX. Informed voluntary consent declaration form**

 I have read/was read to me the participant information sheet

 I have clearly understood the purpose of the research, the procedures, the risks and benefits, issues of confidentiality, the rights of participating and the contact address for any queries

 I have been given the opportunity to ask questions for things that may have been unclear or

 I was informed that I have the right to withdraw from the study at any time or not to answer any question that I do not want to

Therefore, I declare my voluntary consent to participate in this study

 I consent to have a feces sample collected from my child

 I consent to be interviewed

 I consent to a household and other relavant household linked contact sites visit, where samples will be taken

 I consent to provide a sample of my own feces

 I consent to provide a feces samples from my child’s youngest sibling.

I approve samples to be stored and sent abroad for further analysis:  Yes	 No

Name of participant/chold ______________________

Name of caretaker/guardian ____________________ Signature ___________ Date___________

Name of researcher obtaining consent _________________ signature _________ Date_______

Name and signature of witnesses (healthcare facility staff members available at a time)

1. _______________________________________________________

2. _______________________________________________________

2. Please review your reference list to ensure that it is complete and correct. If you have cited papers that have been retracted, please include the rationale for doing so in the manuscript text, or remove these references and replace them with relevant current references. Any changes to the reference list should be mentioned in the rebuttal letter that accompanies your revised manuscript. If you need to cite a retracted article, indicate the article’s retracted status in the References list and also include a citation and full reference for the retraction notice.

― Generally, only one new reference has been cited in this revised manuscript, i.e.., Gobena *et al.* [20]**.** This reference has been newly cited because it contained the details of the sample collection protocol briefed in this study. The cited article has been accepted and approved for publication in SAGE/Environmental Health Insights. DOI: 10.1177/11786302241288167. Furthermore**,** the references that were already cited were checked, and the citation style has been corrected to the journal guidelines.

Authors response to Reviewer's Comments and Questions

Reviewers' comments:
Reviewer's Responses to Questions

**Comments to the Author**
1. If the authors have adequately addressed your comments raised in a previous round of review and you feel that this manuscript is now acceptable for publication, you may indicate that here to bypass the “Comments to the Author” section, enter your conflict of interest statement in the “Confidential to Editor” section, and submit your "Accept" recommendation.

Reviewer #2: (No Response)

Reviewer #3: All comments have been addressed

2. Does this manuscript meet PLOS Global Public Health’s [publication criteria](https://c05y1x9s.r.us-east-2.awstrack.me/L0/https:%2F%2Fjournals.plos.org%2Fglobalpublichealth%2Fs%2Fcriteria-for-publication/1/010f0192227ac801-d62a6718-9ea9-460e-ba30-16d97d288341-000000/ZI1azhMjeA2IMyY7nyvBEA0dIeI=177)? Is the manuscript technically sound, and do the data support the conclusions? The manuscript must describe methodologically and ethically rigorous research with conclusions that are appropriately drawn based on the data presented.

Reviewer #2: Yes

Reviewer #3: Yes

3. Has the statistical analysis been performed appropriately and rigorously?

Reviewer #2: Yes

Reviewer #3: Yes

4. Have the authors made all data underlying the findings in their manuscript fully available (please refer to the Data Availability Statement at the start of the manuscript PDF file)?
The [PLOS Data policy](https://c05y1x9s.r.us-east-2.awstrack.me/L0/https:%2F%2Fjournals.plos.org%2Fglobalpublichealth%2Fs%2Fdata-availability/1/010f0192227ac801-d62a6718-9ea9-460e-ba30-16d97d288341-000000/oqLM7fdKpa6Q8yPpa2hwKsMQhSs=177) requires authors to make all data underlying the findings described in their manuscript fully available without restriction, with rare exception. The data should be provided as part of the manuscript or its supporting information, or deposited to a public repository. For example, in addition to summary statistics, the data points behind means, medians and variance measures should be available. If there are restrictions on publicly sharing data—e.g. participant privacy or use of data from a third party—those must be specified.

Reviewer #2: Yes

Reviewer #3: Yes

5. Is the manuscript presented in an intelligible fashion and written in standard English?
PLOS Global Public Health does not copyedit accepted manuscripts, so the language in submitted articles must be clear, correct, and unambiguous. Any typographical or grammatical errors should be corrected at revision, so please note any specific errors here.

Reviewer #2: Yes

Reviewer #3: Yes

6. Review Comments to the Author

Please use the space provided to explain your answers to the questions above. You may also include additional comments for the author, including concerns about dual publication, research ethics, or publication ethics. (Please upload your review as an attachment if it exceeds 20,000 characters)

**Authors Response to Reviewer comments (**# **Reviewer2)**

Dear Reviewer 2,

We are grateful for your constructive comments and suggestions you provided for the improvement of our manuscript. The authors checked all comments provided and have made corrections accordingly.

**Changes that has been made to the manuscript:**

― The authors have made language editions.

― Specific comments and suggestions given by reviewers were considered, and the authors' responses have been given line by line.

However, some comments were already addressed in the already submitted version of the manuscript, and the authors have only given clarity for such comments and suggestions in this version.

**Response to specific comments and suggestions**

**Reviewer 2:** The manuscript centred on detection of Campylobacter species in under fives with acute diarrhoea and testing for the organism among the family (caretaker) contacts of the child as well as the environment where the diarrheic cases are found including remnant of foods etc. The project takes in so many areas of investigation and tried to establish presence of two major campylobacter species.

The manuscript looks quite a bit voluminous. It will be good if the introduction and discussion could be reduced by discussing more of the results and moderating comparisons with similar works.

**Authors Response**

― We agree that the manuscript seems bulky. Initially, we submitted a version containing bacteriological data and risk factors to PLOS with plans to publish a separate manuscript focusing on the WGS data. However, in light of PLOS's data availability policy and the comments from reviewers, we added the phylogenetic tree analysis and modified both the title and the objective of the study accordingly, which has resulted in a somewhat voluminous manuscript. We have also added two authors to the authorship list based on their contributions to the newly included genomic analysis data.

― In this version, we have revised the manuscript to minimize its size where possible, in response to the comments received. Furthermore, we have worked to reduce the methodology section (**Collection of Exposure Source Samples**) by referencing issues described in recently published articles on other foodborne pathogens from the same project by the same authors.

**Reviewer 2:** Lines 93: remove sample(s)

― Accepted and has already been corrected in the previously revised version of the manuscript (V1).

**Reviewer 2:** Line 102: for the recent exposure details (let’s define the recent e.g was it 2 or 3 days or more?)

― The comment is appreciated and well accepted because it was what should be described. This “recent exposure” was determined by considering the incubation period of the *Campylobacter* infection (usually from hours to 5 days, with the possibility of extension to 10 days).

― It has been corrected and incorporated in the methodology (in the **Sample types and collection procedures)** section**,** with the contextual terminological definitions of “exposure source,” "direct exposure environment," and “indirect exposure environment" used in the study/manuscript.

**Reviewer 2:** Line 134: Sentence should be closed with a bracket. What measures did you put in place to ensure that the stool samples handed over to you by the caretakers of the child really belonged to them?

― The stool samples analyzed in this study were collected by health professionals. Diarrheic stool samples were collected directly from children in collaboration with laboratory technicians at local healthcare facilities.

― Caretakers provided their own stool samples being in a toilet room used at healthcare facilities for the same. Only one person entered the room at a time, and data collectors received the samples at a close distance from the entrance, ensuring there was no means of mixing or cheating for samples. Additionally, caretakers (mainly mothers) were provided voluntary consent after fully informed about the study's objectives and the benefits that the local community (including their family) could obtain from contact’s (caretakers’) stool analysis → input for child health improvement strategy.

― Furthermore, this is not the first study of its kind in healthcare facilities, and this method is applicable across the globe that adults responsibly provide their own stool samples, which is scientifically acceptable. In fact, this study is part of FOCAL (Foodborne Disease Epidemiology, Surveillance, and Control in African Low- and Middle-Income Countries), and many articles of the same protocol have been published. These stool samples were not only analyzed for *Campylobacter* but other FBPs like *E. coli*, Salmonella, etc.; for instance, our article <https://pubmed.ncbi.nlm.nih.gov/39187763/> was produced from the same project/protocol.

**Reviewer 2:** Line 211: A little confusion here. You mean the child’s stool was collected at health care facilities or are remnants of the stool collected at the health care facility for testing prior to treatment.

― Of course, child stool samples were collected at healthcare facilities (the confusion may be due to grammatical errors, which may give different meanings).

**Clarity:** We believe that both sentences have the same meaning. This means the child stool samples were not sourced from households or other locations; rather, they were collected at healthcare facilities prior to treatment. meaning that only children with acute diarrhea—who sought medical care—were enrolled in this study.

In these cases, stool examinations, along with other diagnostic parameters, were ordered by physicians or clinicians. Therefore, stool samples analyzed in this study were collected at the same time when laboratory technicians also received the child’s stool for examination.

**Reviewer 2:** Line 234: Please decide on whether to use one decimal place or two to make the figures uniform.

― The comment is accepted and has been corrected. Except in the tables or figures, one decimal place was used throughout the manuscript.

**Reviewer 2:** Line 266: Please let the audience know what constituted the exposure environment from which the graph was derived. Is it the same thing as items on lines 118-120?

― Yes,

Clarity: Exposure environment is defined in the methodology (in the **Sample types and collection procedures)** section of the already submitted revised version of the manuscript (v1).

― In this study, the term “exposure source” indicates any accessible sample sources that were …... These include food, animal, wastewater, and water sources. Furthermore, in the data analysis section, it was described that “The monthly *Campylobacter* occurrence rate was calculated by considering the number of *Campylobacter* isolates obtained in a given month and the total number of samples analysed in the laboratory in that particular month.”

Line 324: Please recast the statement from However (10) reported 9.3% Campylobacter species..........to e.g. Other workers reported 9.3% Campylobacter species involvement ……………… (10)

― Accepted and has been corrected.

**Reviewer 2:** Lines 384-385: Please change to …… of previous studies conducted by other workers which gave 56.5%f (12) and 14.6% (10).

― Accepted and has been corrected

**Reviewer 2:** Line 416: may lead to an enhancement?? In what way please?

― This part was already revised and submitted in the revised version of the manuscript (v1).

**Authors Response to Reviewer comments (**# **Reviewer3)**

**Reviewer #3**: I think authors not necessory to show the pictures of the The spectrophotometric readings for DNA purity obtained from some samples. I recomonded removing those and just mentiond the DNA concentration.

― Accepted has been removed. In fact, this was done in the already revised and submitted version of the manuscript (v1).
